# Wearable robotic exoskeleton for gait reconstruction in patients with spinal cord injury: A literature review

**DOI:** 10.1016/j.jot.2021.01.001

**Published:** 2021-03-01

**Authors:** Koki Tan, Soichiro Koyama, Hiroaki Sakurai, Toshio Teranishi, Yoshikiyo Kanada, Shigeo Tanabe

**Affiliations:** a Graduate School of Health Sciences, Fujita Health University, Toyoake, Aichi, Japan; b Faculty of Rehabilitation, School of Health Sciences, Fujita Health University, Toyoake, Aichi, Japan

**Keywords:** Gait reconstruction, Paraplegia, Tetraplegia, Wearable robotic exoskeleton

## Abstract

**Objectives:**

Wearable robotic exoskeletons (WREs) have been globally developed to achieve gait reconstruction in patients with spinal cord injury (SCI). The present study aimed to enable evidence-based decision-making in selecting the optimal WRE according to residual motor function and to provide a new perspective on further development of appropriate WREs.

**Methods:**

The current review was conducted by searching PubMed, Web of Science, and Google Scholar for relevant studies published from April 2015 to February 2020. Selected studies were analysed with a focus on the participants’ neurological level of SCI, amount of training (number of training sessions and duration of the total training period), gait speed and endurance achieved, and subgroup exploration of the number of persons for assistance and the walking aid used among patients with cervical level injury.

**Results:**

A total of 28 articles (nine using Ekso, three using Indego, ten using ReWalk, one using REX, five using Wearable Power-Assist Locomotor) involving 228 patients were included in the analysis. Across all WREs, T6 was the most frequently reported level of SCI. The amount of training showed a wide distribution (number of training sessions: 2–230 sessions [30–120 min per session]; duration of the total training period: 1–24 weeks [1–5 times per week]). The mean gait speed was 0.31 m/s (standard deviation [SD] 0.14), and the mean distance on the 6-min walking test as a measure of endurance was 108.9 m (SD 46.7). The subgroup exploration aimed at patients with cervical level injury indicated that 59.2% of patients were able to ambulate with no physical assistance and several patients used a walker as a walking aid.

**Conclusion:**

The number of cervical level injury increased, as compared to the number previously indicated by a prior similar review. Training procedure was largely different among studies. Further improvement based on gait performance is required for use and dissemination in daily life.

**The translational potential of this article:**

The present review reveals the current state of the clinical effectiveness of WREs for gait reconstruction in patients with SCI, contributing to evidence-based device application and further development.

## 1 Introduction

Spinal cord injury (SCI) is a traumatic event with an incidence of 3.6–195.4 cases per million worldwide [1], 17,810 cases per year in 2020 in the United States [2], and approximately 5000 cases per year and approximately 200,000 in total in 2010 in Japan [3]. Data from the US National Spinal Cord Injury Statistical Center indicated that more than half of patients with SCI have cervical level injury, with C5 level injury constituting the largest category in 2015 worldwide, followed by thoracic level injury [2]. Similarly, in Japan, 75% of cases have injury at the cervical level, whereas the remaining 25% have injury at the thoracolumbar level [4]. Approximately 17% of patients with cervical SCI who have complete motor paralysis lose voluntary neural control below the lesion level [5].

Despite undergoing intensive rehabilitation, functional recovery has been commonly incomplete. Most SCI patients have long-term motor disability. The risk of various secondary complications is caused by daily living on wheelchair that requires long-term medical treatment (e.g., joint contraction, pressure sores, and osteoporosis), and patients cannot achieve independent standing and walking even after rehabilitation [6].

Patients with motor-complete SCI usually use a wheelchair to move toward a destination in their activities of daily living. Wheelchair is a useful mobility device for patients with SCI because of its high energy efficiency [7]. Nevertheless, sitting on a wheelchair for a long period leads to various medical (e.g. joint contraction, pressure sores, osteoporosis) [8,9] and psychosocial problems consequent to a relatively low eye level [10,11]. Standing and gait reconstruction to prevent the development of these problems in such patients has been a key issue in rehabilitation medicine [12]. Medical point of view, previous studies showed that an upright position reduces the risk of lower-extremity joint contractures, osteoporosis, spasticity, bedsores, and oedema in patients with SCI [13,14].

As an early developed device for standing and gait reconstruction, knee–ankle–foot orthosis was proposed for individuals with motor-complete low-level injury or motor-incomplete SCI, and hip–knee–ankle–foot orthosis and reciprocating gait orthosis were subsequently developed [[15], [16], [17], [18]]. Previous studies have shown that the application of these orthoses to patients with SCI (T6–T12, American Spinal Injury Association impairment scale [AIS] B) can improve their walking performance, including walking speed and endurance [19]. However, these orthoses are limited by their high metabolic demand [[20], [21], [22], [23]].

Some wearable robotic exoskeletons (WREs) have recently been developed for gait reconstruction in patients with motor-complete SCI [24,25]. WREs offer patients with SCI with the opportunity to comfortably walk at home and in the community by moving both of their paretic legs in a reciprocal stepping pattern [26,27]. While their development has advanced since the 1960s, their practical application has not been attained owing to the lack of sufficient control technology and actuator performance [28]. Advancements in related robotic technology have greatly facilitated the transition from the research stage to the practical stage for WREs. Arazpour et al. and Yatsuya et al. reported that the gait speed and endurance of patients with SCI using WREs were superior to those using reciprocating gait orthosis and hip–knee–ankle–foot orthosis [29,30]. Asselin et al. showed that oxygen consumption and heart rate during gait were lower with WRE than with conventional orthosis [31].

Some previous reports have reviewed clinical trials that investigated gait reconstruction with WREs [26,27,[32], [33], [34], [35], [36]]. These reports focused on the device’s features, clinical efficacy, and participants’ neurological level of SCI. Chen et al. summarized that WREs are mainly developed in three different types of applications: gait rehabilitation for providing more effective rehabilitation in patients with gait disorder, human locomotion assistance for reconstruction of the ability to stand up, to sit down, and to walk independently, and enhancing physical strength and endurance to easily perform long hours of hard work [32]. For performance assessment methods in clinical efficacy, Huo et al. classified these methods into three categories in the previous review: the gait endurance as metabolic energy expenditure with or without using WREs, the gait performance such as kinematic variables, temporal–spatial gait variables, and physiological cost variables, and the muscular activity analysis to activate the neuromuscular function with paralyzed lower limbs [33]. Contreras-Vidal et al. reviewed relevant articles published until December 2015 [34]. In their review, injuries at the T10 level were represented in 45.4% of all included articles and five studies enrolled patients with cervical level injury [34]. Five out of seven articles reported improvement in gait performance as a result of WRE use, whereas two studies could not yield positive results. Nonetheless, to the best of our knowledge, there has been no review comparing WREs in relation to the neurological level of SCI since 2015. With respect to scientific publications, this is a fast-paced advancing field; hence, a new literature review is warranted. Additionally, differences in the amount of training and in the gait performance achieved among WREs are unclear, and there has been no review involving the number of persons for assistance and the walking aid used, even though these points are an important perspective for training among patients with cervical level injury.

The current review had four study objectives. First, this review aimed to summarize clinical trials on WREs published from 2015 to 2020 in order to clarify the recent distribution of participants’ neurological level of SCI. Second, this review also sought to identify the amount of training in terms of the number of gait training sessions and duration of the total training period for each robot. Third, this review aimed to compare gait performance among WREs with respect to the gait speed and endurance achieved. Finally, this review sought to conduct a subgroup exploration of the number of persons for assistance and the walking aid used among patients with cervical level injury who commonly present with poor trunk function. The findings might not only enable evidence-based decision-making in selecting the optimal WRE according to the residual motor function of patients with SCI but also provide a new perspective on further development of appropriate WREs.

## 2 Materials and methods

### 2.1 Literature search

The present review was conducted in accordance with the Preferred Reporting Items for Systematic Reviews and Meta-Analyses (PRISMA) and was performed by searching PubMed, Web of Science, and Google Scholar for relevant papers published from April 2015 to February 2020. The following keywords were used for the literature search: ‘spinal cord injury’, ‘paraplegic’, ‘paraplegia’, ‘tetraplegic’, ‘tetraplegia’, ‘lower limb paralysis’, ‘lower-extremity paralysis’ associated with ‘exoskeleton’, ‘powered exoskeleton’, ‘robotics’, ‘robot’, ‘assisted gait’, ‘robotic ambulation’, ‘robotic gait’, ‘robotic assisted gait’, ‘gait training’, ‘locomotor’, ‘locomotor treatment’, and ‘locomotion’. Subsequently, a manual search based on the reference lists of the included papers and other relevant meta-analyses was conducted in May 2020.

### 2.2 Study selection

**Appendix Fig. 1:**
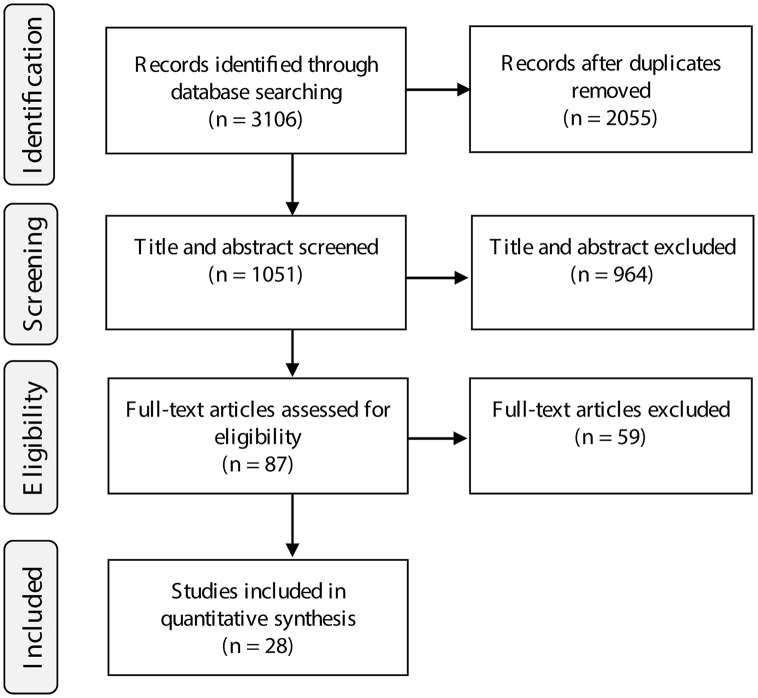
Analysis procedure and study results during Preferred Reporting Items for Systematic Reviews and Meta-Analyses (PRISMA) phases: a flowchart of selection process based on the inclusion/exclusion criteria.

A flow diagram of study identification and selection is shown in Appendix Fig. 1. The literature search undertaken yielded 3106 records. First, duplicate articles were manually removed, and review articles and conference proceedings were excluded. Second, screening by examining the titles and abstracts of studies resulted in the exclusion of articles dealing with an unrelated topic. In the remaining articles, the full text was assessed to determine eligibility.

In the present review, (1) selected articles that did not mention any robot devices, described an unclear robot device, and had an unrelated topic; (2) papers mentioning upper-body exoskeletons only; and (3) articles mentioning only EMG hybrid exoskeletons requiring volitional lower-extremity contraction or training limited to a treadmill were excluded from the analysis.

### 2.3 Outcome and data analysis

Selected articles were analysed with a focus on the participants’ neurological level of SCI, amount of training (number of training sessions and duration of the total training period), gait performance (speed and endurance) achieved, and subgroup exploration of the number of persons for assistance and the walking aid used.

With respect to the participants’ neurological level of SCI, data of patients with motor-complete SCI (AIS A and B) were extracted. The highest level of SCI was selected when there was left-right asymmetry or when the injury was extensive. Following classification of participants according to their neurological level of SCI, the number of patients for the total and individual robots was calculated. The proportion of patients for the individual robots was also calculated according to lesion level (i.e. cervical, upper thoracic, lower thoracic, and lumbar).

The amount of training (number of training sessions [along with minutes per session and sessions per week] and duration of the total training period) was calculated for all patients in the selected studies. The term ‘amount of training’ described in each paper had three different definitions—namely, (1) that all participants performed gait training with a predetermined period; (2) that each participant performed training with a sufficient period on a case-by-case basis; and (3) that participants continued gait training even after completing a sufficient training period to achieve the highest gait performance for each participant. In the third case, the total training period referred to the time from the start of training using WREs until reporting. Furthermore, if there was a clearly stated practice procedure, the content of that procedure was confirmed.

The gait performance (gait speed and endurance) was calculated for patients with motor-complete SCI (AIS A and B) in selected studies. If explicitly described, the value of the gait speed described was used. In the absence of such data, it was calculated using the 10-m walking test. As for gait endurance, two outcome measures were used: 6-min walking test (6MWT) and continuous walking time. The mean and standard deviation (SD) of gait performance were calculated for the total and individual robots. Additionally, a histogram of gait performance was constructed to confirm each distribution.

With respect to the subgroup exploration of the number of persons for assistance and type of walking aid used during walking, data of patients with motor-complete or motor-incomplete (AIS A to D) cervical level injury were extracted from selected studies. If the participants walked under supervision or without physical assistance, the number of persons was counted as zero. If the participants required assistance by touching their body, the number of persons was counted, as needed. Both pieces of information were confirmed for each robot.

## 3 Results

A total of 28 studies [12,27,30,[37], [38], [39], [40], [41], [42], [43], [44], [45], [46], [47], [48], [49], [50], [51], [52], [53], [54], [55], [56], [57], [58], [59], [60], [61]] involving 228 patients were eventually included in the present review: Ekso® (Ekso Bionics, Richmond, CA, USA) was used in nine studies, Indego™ (Parker Hannifin Corp., Cleveland, OH, USA) in three studies, ReWalk™ (ReWalk Robotics Inc., Marlborough, MA, USA) in ten studies, Wearable Power-Assist Locomotor (WPAL; ASKA Corp., Aichi, Japan) in five studies, and REX (Rex Bionics Ltd., Auckland, New Zealand) in one study. The main features of the WRE were showed in Table Appendix 1. Of the 228 patients, 185 had motor-complete SCI (AIS A or B), whereas 43 had motor-incomplete SCI (AIS C or D).

### 3.1 Participant’s neurological level of SCI

**Figure 1:**
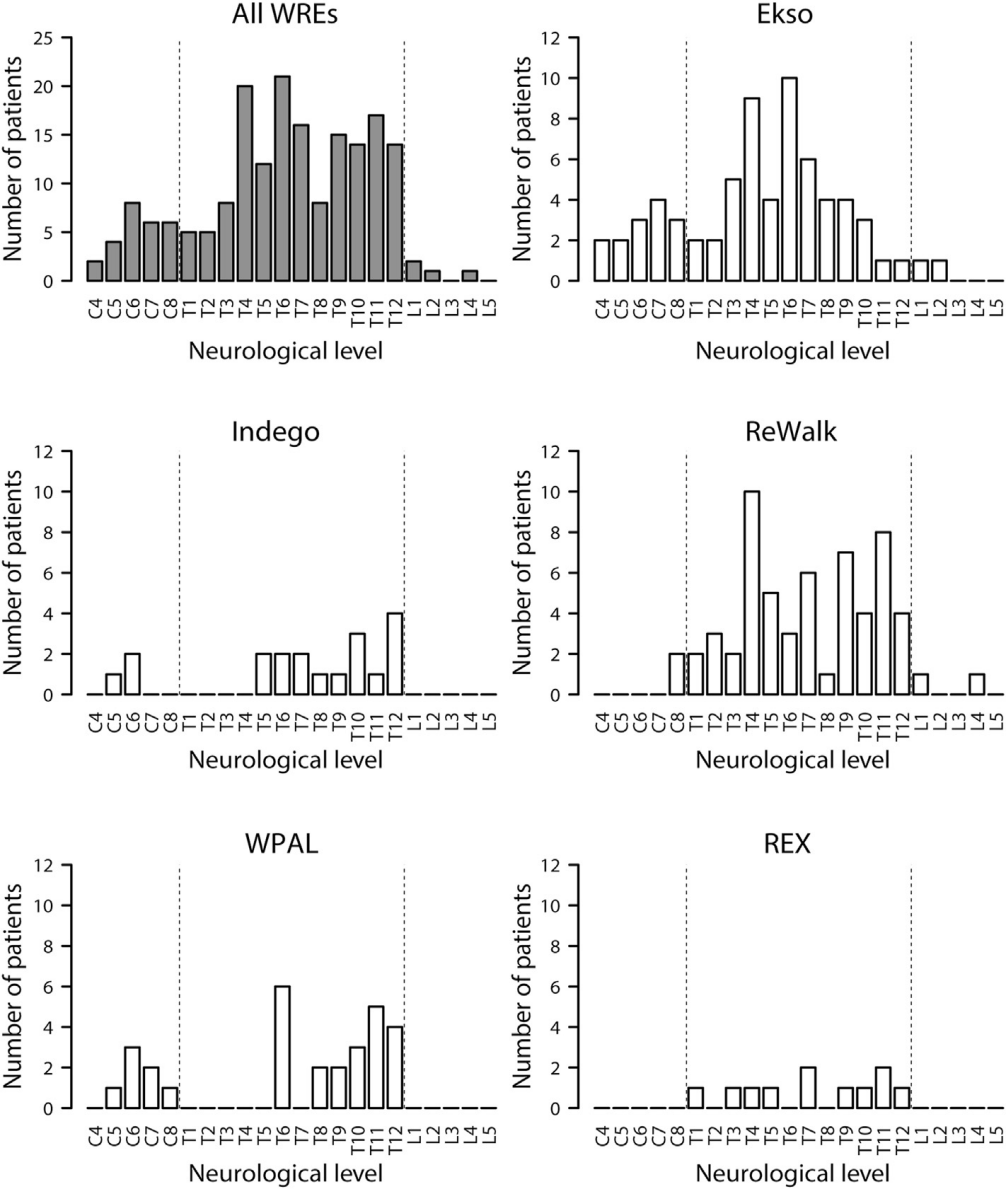
Number of patients according to the neurological level. The total number of patients according to neurological level is shown. WRE, Wearable robotic exoskeleton; WPAL, Wearable Power-Assist Locomotor.

**Appendix Fig. 2:**
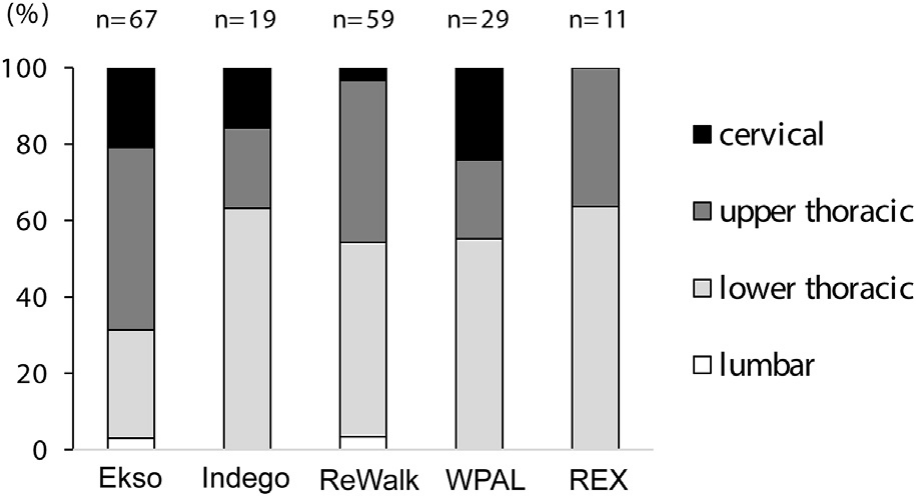
Proportion of patients according to the lesion level.

The number of patients when the participants were classified according to neurological level of SCI is presented in Fig. 1, whereas the proportion of patients according to lesion level is presented in Appendix Fig. 2. Neurological levels ranged from C4 to L4, with T6 being the most frequently reported level of SCI among all devices (11.3% of all cases and 39.2% of all articles). Most studies included patients with thoracic level injury, and 16 out of 28 studies (57.1%) included patients with cervical level injury. A total of 39 participants had cervical level injury. None of the studies recruited patients with sacral level injury. In this review, most participants had motor-complete SCI (AIS A or B), with patients with motor-complete cervical level injury accounting for 13.5% of all motor-complete SCI cases and 64.1% of all cervical SCI cases. The time since the occurrence of an injury ranged from 3 weeks to 31 years. Most studies recruited patients with chronic (>6 months) SCI. One study included a patient with SCI in the acute phase [41].

### 3.2 Amount of training

Table Appendix 2 presents the amount of training for each study. Seven articles did not describe the amount of training. The amount of training was described separately for the number of sessions (along with minutes per session and sessions per week) and duration of the total training period. The results indicated large differences in the amount of training. Across all studies, the maximum number of sessions was 230, whereas the minimum number was two. The duration of one session ranged from 30 to 120 ​min, and there were one to five sessions per week. Additionally, there was considerable variation in the duration of the total training period, ranging from 1 to 24 weeks.

Basic training procedures were mentioned for two WREs. Ekso has four walk modes with different level of difficulty—namely, ‘First Step mode’, ‘Active Step mode’, ‘Pro Step mode’, and ‘Pro Step Plus mode’ [62]. In the ‘First Step mode’, the supervisor uses a push button controller to generate a step after appropriate weight shift. In the ‘Active Step mode’, patients push the controller buttons on crutches or walker as a trigger for Ekso’s stepping motion. In the ‘Pro Step mode’, the step is automatically generated when the device detects that the patient has achieved appropriate weight shift. In the ‘Pro Step Plus mode’, stepping motion is performed upon simultaneous detection of both appropriate weight shift and voluntary contraction in the paretic legs. WPAL employs the following five-step training method [63]: (1) stepping exercise within parallel bars, (2) gait exercise within parallel bars, (3) gait exercise on a treadmill, (4) gait exercise using a walker with a safety harness, and (5) gait exercise using a walker without a safety harness.

### 3.3 Gait performance

**Figure 2:**
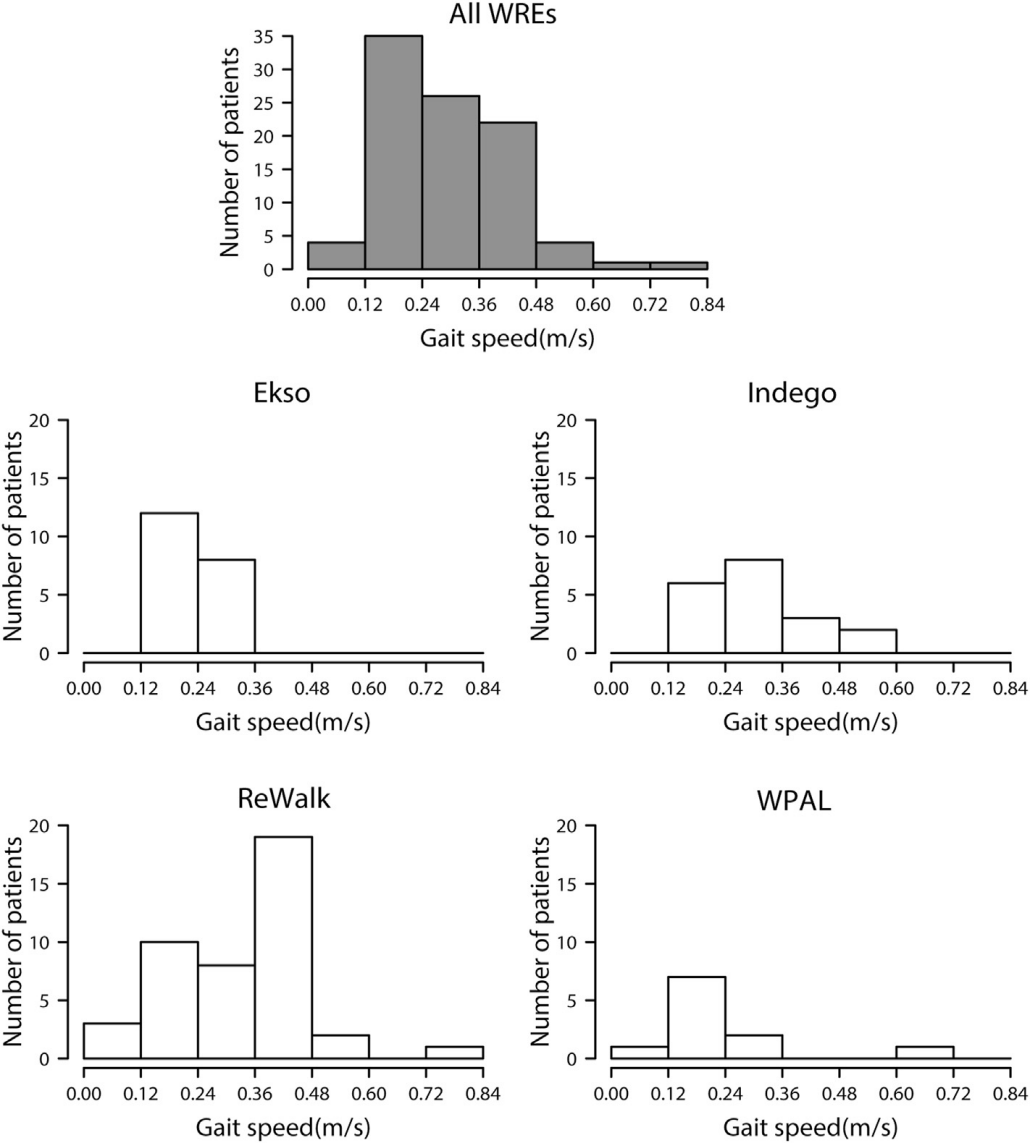
Number of patients according to gait speed. The number of gait speed classes was determined based on Sturges’ histogram rule. WRE, Wearable robotic exoskeleton; WPAL, Wearable Power-Assist Locomotor.

The number of patients when the participants were classified according to gait speed is presented in Fig. 2. Gait speed was measured in 20 patients using Ekso, 19 patients using Indego, 43 patients using ReWalk, and 11 patients using WPAL. The mean gait speed for all WREs was 0.31 ​m/s (SD 0.14); specifically, the mean gait speed was 0.24 ​m/s (SD 0.06) for Ekso, 0.32 ​m/s (SD 0.10) for Indego, 0.33 ​m/s (SD 0.15) for ReWalk, and 0.24 ​m/s (SD 0.20) for WPAL. The fastest speed was 0.83 ​m/s, which was recorded in a patient with motor-complete SCI who had neurological level of L1 using ReWalk. The slowest speed was 0.03 ​m/s, which was recorded in a patient with motor-complete SCI who had neurological level of C6 using WPAL.

**Figure 3:**
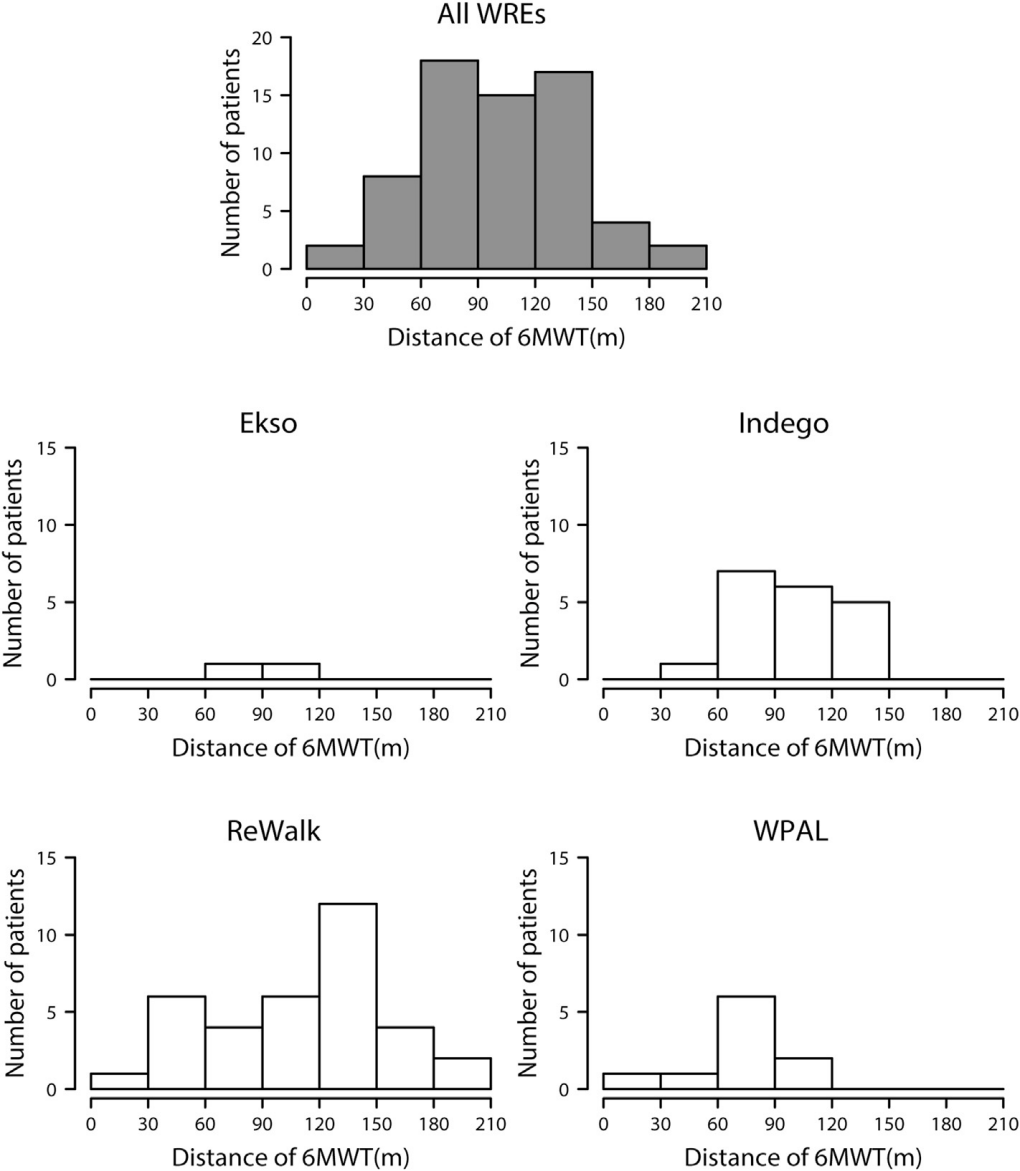
Number of patients according to gait distance on the 6-min walking test (6MWT). The number of gait distance classes was determined based on Sturges’ histogram rule. WRE, Wearable robotic exoskeleton; WPAL, Wearable Power-Assist Locomotor.

The number of patients when the participants were classified according to gait distance on the 6MWT is presented in Fig. 3. Gait distance on the 6MWT was measured in two patients using Ekso, 19 patients using Indego, 35 patients using ReWalk, and ten patients using WPAL. The mean gait distance for all WREs was 108.9 ​m (SD 46.7); specifically, the mean gait distance was 84.7 ​m (SD 18.0) for Ekso, 94.9 ​m (SD 27.5) for Indego, 115.9 ​m (SD 47.1) for ReWalk, and 71.0 ​m (SD 26.2) for WPAL. The longest gait distance was 209 ​m, which was recorded in a patient with motor-complete SCI who had neurological level of T4 using ReWalk. The shortest gait distance was 11.8 ​m, which was recorded in a patient with motor-complete SCI who had neurological level of C6 using WPAL.

In two WREs, continuous walking time was measured instead of the gait distance on the 6MWT, and 25 and 15 patients used Ekso and WPAL, respectively. The mean time for both WREs was 33.8 ​min (SD 20.8); specifically, the mean time was 40.5 ​min for Ekso and 22.3 ​min for WPAL. The longest walking time using each robot was 94.0 ​min, which was recorded in a patient with motor-complete SCI who had neurological level of T8 using Ekso, and 84.0 ​min, which was recorded in a patient with motor-complete SCI who had neurological level of T10 using WPAL. The shortest walking time using each robot was 19.0 ​min, which was recorded in a patient with motor-complete SCI who had neurological level of C6 using Ekso, and 4.7 ​min, which was recorded in a patient with motor-complete SCI who had neurological level of C7 using WPAL.

As an index of gait performance, the timed-up and go test was used in four studies (Ekso 1, ReWalk 2, and REX 1). These mean scores were 28.3 ​s for Ekso, 58.3 ​s for ReWalk, and 313.0 ​s for REX, respectively. As temporal–spatial parameters of gait with Ekso and WPAL, some studies reported mean step length (12.8 ​cm for Ekso, and 19.6 ​cm for WPAL). For Exso, the mean swing time and the number of steps were also reported (1.25 ​s and 889 steps/session).

### 3.4 Subgroup exploration in patients with cervical level injury

Table 1 shows the number of persons for assistance and the type of walking aid used during walking among patients with cervical level injury according to WREs. In the present review, 39 participants had cervical level injury. The number of persons for assistance was clearly specified in 27 participants: seven using Ekso, three using Indego, five using ReWalk, five using REX, and seven using WPAL. For each WRE, the mean number of persons for assistance was 0.71 for Ekso, 1.00 for Indego, 0 for ReWalk, 1.00 for REX, and 0.28 for WPAL. After training using each WRE, 59.2% of patients with cervical level injury were able to ambulate with no physical assistance.

**Table 1:** Number of assistants and walking aid used among patients with cervical level injury for each robot.

Robot	Number of persons for assistance			Walking aid				
	Zero	Single	Multiple	FC	W	RP	FW	RW
Ekso	4	1 (1)	2	2 (1)	4	1	1 (1)	1
Indego	—	3	—	—	—	3	—	—
ReWalk	5 (4)	—	—	5 (4)	—	—	—	—
WPAL	5	2	—	—	5	—	—	—
REX	2 (2)	1 (1)	2 (2)	—	—	—	—	—

If the participants walked under supervision or without physical assistance, the number of persons was counted as ‘zero’. If a certain person provided assistance to the participants by touching their body, it was counted as ‘single’, and if assistance was provided by two or more people, it was counted as ‘multiple’.

FC, forearm crutches; W, four-wheeled walker; RP, rolling platform walker; FW, front-wheeled walker; RW, rigid walker; WPAL, Wearable Power-Assist Locomotor.

The number of patients with motor-incomplete SCI is shown within parentheses.

The type of walking aid used was clearly specified in 22 participants: nine using Ekso, three using Indego, five using ReWalk, and five using WPAL. Commonly, patients with cervical level injury used a walker. However, REX did not require the use of a walking aid. Two participants (one with Ekso and one with ReWalk) with motor-complete SCI used forearm crutches to walk. As for Ekso, the neurological level of the patient was C8 (AIS B); the patient continuously walked at a gait speed of 0.21 ​m/s for 62 ​min without physical assistance. As for ReWalk, the neurological level of the patient was C8/T8 (AIS A); the patient was able to walk without physical assistance (gait speed: 0.42 ​m/s).

## 4 Discussion

The current review provided insight about the participants’ neurological level of SCI, amount of training, and gait performance. Furthermore, a subgroup exploration of the number of persons for assistance and the type of walking aid used among patients with cervical SCI was conducted.

### 4.1 Participant’s neurological level of SCI

In the present review, T6 was the most frequently reported level of SCI among all devices (11.3% of all cases and 37.9% of all articles). A previous review conducted by Contreras-Vidal et al. showed that injury at the T10 level was represented in 45.4% of the included articles [34]. The difference suggests that the number of patients with higher level of SCI who are attempting to achieve gait reconstruction has been increasing. Patients with cervical level injury were enrolled in five out of 22 articles (22.7%) in the previous review [34]; in comparison, these patients were included in 16 out of 28 articles (57.1%) in the present review. Gait reconstruction for patients with cervical level injury is a recent important topic, considering that more than half of patients with SCI have cervical level injury, particularly at the C5 level (the largest category) [2].

### 4.2 Amount of training

The present review revealed that with respect to the amount of training, there was a large difference in the number of sessions and duration of the total training period, even among articles using the same WREs. To reduce the variability in the amount of training and standardize basic training procedures, future studies exploring refinements in training procedures with accurate prediction of the final outcome of gait performance using WREs should be performed. Studies with a large sample size that employ decision tree analysis may be needed to predict the final outcome. A previous study reported that neurological level of injury, age, residual motor function in the upper limbs, and spasticity affect the final gait performance of patients with SCI using conventional orthosis [17].

### 4.3 Gait performance

The mean and fastest gait speeds were 0.31 ​m/s and 0.83 ​m/s, respectively. Patients with SCI might not be able to use WREs outdoors and in crowded cities; however, their use in limited communities and home environments is conceivably sufficient. A meta-analysis investigating the normal gait speed in elderly participants aged 80–99 years reported a mean gait speed of 0.95 ​m/s [64]. Another study evaluating gait speed for pedestrian crossing conditions in elderly participants reported a mean gait speed of 1.44 ​m/s [65].

The mean and longest distances on the 6MWT as a measure of endurance were 108.9 ​m and 209 ​m, respectively. These results suggest that the use of WREs for patients with SCI might be conceivably sufficient in limited communities and home environments. Enright and Sherrill reported at least 200 ​m on the 6MWT for elderly adults aged 70–80 years [66]. In another previous study, walking with robots was equivalent to light exertion, and patients felt that they could sustain walking for extended durations [26].

With respect to the comparison of gait speed and gait distance among WREs, participants using WPAL tended to walk with a slower gait speed and a shorter gait distance for a limited period (0.24 ​m/s and 71.0 ​m on the 6MWT, respectively). These findings suggest that the structure of robotic hip joint system in WREs might affect the gait speed and gait distance. WREs can be categorized into two types according to the position of the robotic hip joint: bilateral external joint type (lateral joint system; for example, Ekso, Indego, ReWalk, and REX) and medial single joint type (medial joint system; for example, WPAL). The robotic hip joint in the lateral joint system is in the same position as the human joint axis, whereas the robotic hip joint in the medial joint system is under the perineum to achieve the advantage of standing stability and wheelchair compatibility. The medial robotic hip joint in the WPAL has a structure with an anteroposterior curving slide mechanism to move the virtual rotation centre of the robotic hip joint closer to the centre of the physical joint [67]. This structure may limit the stride length, consequently limiting the gait speed and gait distance within a limited period of time. When a novel structure of the medial joint system is invented, this limitation may be removed.

### 4.4 Subgroup exploration in patients with cervical level injury

For patients with cervical level injury, the mean number of persons for assistance was 0.71 for Ekso, 1.00 for Indego, 0 for ReWalk, 1.00 for REX, and 0.28 for WPAL. This result might have been influenced by the difference in the level of residual motor function below the neurological level of injury. The mean number of persons for assistance was zero for ReWalk because four out of five participants using ReWalk had motor-incomplete SCI. In comparison, that for Ekso, Indego, and WPAL ranged from 0.28 to 1.00 because the number of participants with motor-incomplete SCI was only one out of six for Ekso, zero out of three for Indego, and zero out of seven for WPAL. However, the mean number of persons for assistance was 1.00 for REX, although all participants (5 out of 5) had motor-incomplete SCI. While the study did not report any clear reason, one of the reasons might be that participants using REX have a relatively high level of SCI. In particular, three out of five participants had C4 level motor-incomplete SCI.

Most patients with motor-complete cervical level injury often use a walker as a walking aid. In particular, patients with motor-complete SCI at the C4–C6 level used a walker with Ekso and WPAL. Patients with motor-complete SCI at the C8 level and motor-incomplete SCI at the C6 level used forearm crutches with Ekso and ReWalk. These results might have been affected by the neurological level and level of residual motor function. Patients with motor-complete SCI at the C6 level or higher exhibit no elbow extension strength to maintain an upright posture during standing and gait. The stability of walkers is superior to that of forearm crutches. In contrast, patients with motor-complete SCI at the C7 level or lower show residual motor function in the upper extremity for full elbow joint extension to maintain an upright position and hold forearm crutches during gait.

### 4.5 Selecting the optimal WRE

The most important perspective in selecting the optimal WRE for the SCI patients is suitability between characteristics resulting from robot’s structure and patient’s residual motor function and severity of spasticity. For Ekso, ReWalk, and Indego, these are similar structures of both hip joints located at the lateral side of the lower limb. This lateral hip joint system allows a large step length due to a wide range of the hip joint. SCI patients with higher gait performance (e.g., thoracic SCI) might be better to choose Ekso, ReWalk, and Indego. However, Indego may be difficult to use in patients with severe ankle plantar flexor spasticity because of a lack of actuator at the ankle joint. A previous study reported that 53% of SCI patients have severe spasticity [68]. Patients with poor standing balance (e.g., cervical SCI) might be better served by choosing WPAL for gait reconstruction. WPAL has a high standing stability due to a rigidly linked medial hip joint located between both legs, and the user can stand without hand support [30]. For patients with severe motor function of the upper limb, REX might be a better option. REX has a system in which both the lower extremity and trunk are firmly and mechanically fixed to provide automatically suitable balance control during standing and gait.

### 4.6 Limitations

The current review included articles published from 2015 to 2020, with the most novel study in the review being an article published in 2020. Nonetheless, robotic rehabilitation is a fast-paced advancing field with respect to scientific publications and development speed, and continuous review is therefore warranted.

We have summarized these WREs with a focus on clinical trials for SCI patients. However, there are some robots at the developmental phase. CUHK-EXO [69] and MINDWALKER [70] suggest that clinical trials on patients with SCI will be conducted in the near future. With the development of regenerative medicine, WRE using surface EMGs (Hybrid Assistive Limb® [HAL®], CYBERDYNE Inc., Ibaraki, Japan) have possibility of clinical trial on SCI patients with complete motor paralysis.

There are no various evaluations except for the 10- and 6-m walk test. The temporal–spatial parameters of gait (e.g., step length, step width, and dabble support time) and kinematic gait analysis (e.g., ground reaction forces and gait symmetry) are important for the future development of WREs for gait reconstruction in patients with 10.13039/100012102SCI. In addition, there were no reports of subjective perceptions that adequately helped to choose WREs based on the selected studies of the present review. Subjective perception (attitude toward technology, acceptability, usability, and satisfaction) would be conducted to accumulate further evidence in the future.

The present review focused on the neurological level of injury (injury severity) and gait reconstruction (rehabilitation effect) in SCI patients with complete motor paralysis. However, the impact of injury severity on rehabilitation effect is medically (e.g., glycometabolism, intestinal motility, and autonomous nerve) important. Future reviews on the medical rehabilitation effect of gait reconstruction with a robot are needed.

The WREs are often used for gait reconstruction in SCI patients. Since the maximum training period was 24 weeks among the included papers in the present review, the accumulation of further evidence is necessary to clarify the effect of long-term use for daily life. For the use of the robot in daily life, the currently available exoskeletons have a limitation of wearability (easy to wear and carry around). Tanabe et al. reported that alternate usage with a wheelchair is an essential requirement for mobility in patients’ daily lives because of its low metabolic demands and high energy efficiency [71]. Previous studies have reported that the mean wearing time was 10 ​min in Ekso [41] and 8 ​min in Indego [46]. Additionally, the waterproof property of WREs was not clarified in the present review. Hansen et al. reported that a total of 20% of the participants with tetraplegic or paraplegic experienced incontinence daily [72]. Development of WREs with waterproof would be necessary for use in daily life.

## 5 Conclusion

A total of 28 studies with five WREs met the inclusion criteria for review. While more than half of patients with SCI had cervical level injury, the number of studies involving patients with cervical SCI was small. However, there was a report that even patients with cervical SCI could walk without assistance. Further studies comparing multiple robotic devices in the same patients with SCI may be necessary to clarify the characteristics and advantages of WREs.

## Funding

This review was supported by 10.13039/501100001691JSPS KAKENHI Grant Number 19K19810.

## Declaration of competing interest

The authors have no conflicts of interest relevant to this article.

## Figures and Tables

**Appendix Table 1:** the main features of the WRE.

WRE	Ekso	Indego	ReWalk	WPAL	REX
Degree of freedom (Active joint)	H/K/A	H/K	H/K/A	H/K/A	H/K/A
Height limit (cm)	158–188	155–191	160–190	145–180	146–195
Weight limit (kg)	100	113	100	80	100
Control option	E/B/W	E/B/W	E/W	B	B

Degree of freedom: H, hip joint; K, knee joint; A, ankle joint; Control option: E, external operator; B, user-operated via buttons; W, user-operated via buttons

**Appendix Table 2:** Training protocol by each study.

Robot	Author	Year	Duration of one session (min)	Total number of sessions	Sessions per week	Duration of training (week)
Ekso	Kyle McIntosh	2019	60	25	3	—
	Carsten B. Baunsgaard	2018	60	24	3	8
	Carsten B. Baunsgaard	2018	—	—	3	8
	Dany H. Gagnon	2018	45–60	18	—	6–8
	Paolo Milia	2018	45–60	—	5	4
	Read A. Alamro	2018	—	—	—	—
	Robert M. Lester	2018	—	—	—	2
	Ashraf S. Gorgey	2017	60	—	1	10–15
	Allan J. Kozlowski	2015	120	24	1–2	—
Indego	Candy Tefertiller	2018	—	24	3	8
	Clare Hartigan	2015	90	5	—	—
	Nicholas Evans	2015	—	—	—	—
ReWalk	Atif S. Khan	2019	—	—	4	12
	Eleonora Guanziroli	2019	60	14–33	3	8
	Patricia J. Manns	2019	—	—	4	12
	Heidi Muijzer-Witteveen	2018	90	2–28	—	—
	Luciano Bissolotti	2018	—	—	—	—
	Katharina Raab	2016	60	70	5	24
	Pierre Asselin	2016	—	12–54	—	—
	Pierre K. Asselin	2016	60–90	—	3	—
	Thomas Platz	2016	60	—	5	4–5
	Ian Benson	2015	—	20	—	10
WPAL	Ikuko Fuse	2019	—	20–230	—	—
	Shigeo Tanabe	2018	60	12	—	—
	Kanan Yatsuya	2017	30–60	—	3–5	4–12
	Soichiro Koyama	2016	—	—	—	—
	Satoshi Hirano	2015	—	—	—	—

The amount of training is summarized for each article. Those not listed are hyphenated. WPAL, Wearable Power-Assist Locomotor.

## Appendix

Black bar represents cervical level, grey bar indicates upper thoracic injury, light-grey bar indicates lower thoracic injury, and white bar represents lumbar level injury. The total number of patients is 67 for Ekso, 19 for Indego, 59 for ReWalk, 29 for WPAL, and 11 for REX. WPAL, Wearable Power-Assist Locomotor.
